# Metabolically active CD4+ T cells expressing Glut1 and OX40 preferentially harbor HIV during *in vitro* infection

**DOI:** 10.1002/1873-3468.12843

**Published:** 2017-10-11

**Authors:** Clovis S. Palmer, Gabriel A. Duette, Marc C. E. Wagner, Darren C. Henstridge, Suah Saleh, Candida Pereira, Jingling Zhou, David Simar, Sharon R. Lewin, Matias Ostrowski, Joseph M. McCune, Suzanne M. Crowe

**Affiliations:** ^1^ Centre for Biomedical Research Burnet Institute Melbourne Australia; ^2^ Department of Infectious Diseases Monash University Melbourne Australia; ^3^ Department of Microbiology and Immunology University of Melbourne Melbourne Australia; ^4^ CONICET‐Universidad de Buenos Aires Instituto de Investigaciones Biomédicas en Retrovirus y Sida (INBIRS) Buenos Aires Argentina; ^5^ AIDS Cure Research Collaborative Pittsburgh PA USA; ^6^ Cellular and Molecular Metabolism Laboratory Baker IDI Heart and Diabetes Institute Melbourne Australia; ^7^ Monash Micro Imaging Monash University Melbourne Australia; ^8^ Inflammation and Infection Research School of Medical Sciences University of New South Wales Sydney Australia; ^9^ The Peter Doherty Institute for Infection and Immunity The University of Melbourne and Royal Melbourne Hospital Melbourne Australia; ^10^ Division of Experimental Medicine Department of Medicine University of California, San Francisco San Francisco CA USA

**Keywords:** cancer, CD4 T cells, Glut1, HIV, immunometabolism, mTOR, PI3K

## Abstract

High glucose transporter 1 (Glut1) surface expression is associated with increased glycolytic activity in activated CD4+ T cells. Phosphatidylinositide 3‐kinases (PI3K) activation measured by p‐Akt and OX40 is elevated in CD4+Glut1+ T cells from HIV+ subjects. TCR engagement of CD4+Glut1+ T cells from HIV+ subjects demonstrates hyperresponsive PI3K‐mammalian target of rapamycin signaling. High basal Glut1 and OX40 on CD4+ T cells from combination antiretroviral therapy (cART)‐treated HIV+ patients represent a sufficiently metabolically active state permissive for HIV infection *in vitro* without external stimuli. The majority of CD4+OX40+ T cells express Glut1, thus OX40 rather than Glut1 itself may facilitate HIV infection. Furthermore, infection of CD4+ T cells is limited by p110γ PI3K inhibition. Modulating glucose metabolism may limit cellular activation and prevent residual HIV replication in ‘virologically suppressed’ cART‐treated HIV+ persons.

## Abbreviations

**BMI**, body mass index

**cART**, combination antiretroviral therapy

**CFSE**, carboxyfluorescein diacetate succinimidyl ester

**ECAR**, extracellular acidification rate

**EGFP**, enhanced green fluorescent protein

**Glut1**, glucose transporter 1

**HCMV**, human cytomegalovirus

**mTOR**, mammalian target of rapamycin

**PI3K**, phosphatidylinositide 3‐kinases

**RT**, reverse transcriptase

HIV is a retrovirus that relies on the host cell to synthesize large amounts of viral proteins, RNA, and DNA, all of which are essential for viral replication, integration, and dissemination. The energy required to synthesize such biomolecules, as well as the lipid bilayer surrounding the viral genome, is predominantly provided by increased glucose uptake and glycolytic metabolism 1, 2. Recent studies have also shown that other viruses such as human cytomegalovirus (HCMV) induce glycolytic metabolism in host cells to support viral replication 3, 4.

Although activated T cells utilize a variety of energy sources (e.g., glycogen, fatty acids, and amino acids), glucose is considered to be the key energy source for their growth, survival, differentiation, and effector functions 5, 6, 7, 8. Greater metabolic demands are placed on virus‐infected cells. In meeting these imposed challenges, glucose is taken up by glucose transporters and is converted to pyruvate through the glycolysis pathway. Most of the pyruvate is converted to fatty acids for membrane biosynthesis via a process known as citrate cataplerosis 4.

Glucose transporter 1 (Glut1) is the primary glucose transporter on T cells and its cell surface expression is a reflection of the activation state of CD4+ T cells 9, 10. This metabolically activated state makes the cell more permissive to infection by HIV 11. It has been shown that activated immune cells increase surface expression of Glut1 as a functional response to their metabolic requirements 12. Impairment of this process can impact on the survival, differentiation, and immunologic function of T cells 13, 14, and a growing body of evidence supports the notion that immunity and inflammation are dependent on glucose metabolism (reviewed in ref. 15, 16, 17, 18, 19) 20. We have recently shown that increased expression of Glut1 on CD4+ T cells in HIV‐infected individuals is associated with immune activation and CD4+ T cell loss 9. Once activated, T cells undergo a metabolic switch in which glucose is principally metabolized via aerobic glycolysis to support growth, proliferation, and effector functions 21, 22, 23. The transport of glucose by Glut1 across the hydrophobic cell membrane is the first and rate‐limiting step of glucose metabolism. The posttranscriptional regulation of Glut1 is controlled, in part, by the phosphatidylinositide 3‐kinases (PI3K), the serine–threonine kinase, Akt (also known as protein kinase B), and the mammalian target of rapamycin (mTOR). However, Glut1 can also be transcriptionally regulated by the stress‐responsive hypoxia inducible factor 1 alpha 24, 25.

Glucose transporter 1 is a downstream target of the PI3K–Akt pathway and, once activated, this transporter is translocated from the cytoplasm to the cell surface membrane to facilitate increased glucose uptake and metabolism. Class IB PI3K is made up of the p110γ catalytic and p110δ regulatory subunits. These isoforms differ from other PI3K subunits in that they have ‘tissue‐restricted expression’ and are predominantly expressed in white blood cells 26, 27, 28. Several studies have confirmed the role of the PI3K–Akt pathway in Glut1 regulation and in the metabolic regulation of T cells, for example, in response to activating stimuli *in vitro* 5, 22, 29, 30. However, the role of specific isoforms in regulating Glut1 expression on CD4+ T cells in humans remains unclear.

In this study, we used phospho‐flow analysis to evaluate the activation status of the PI3K–Akt pathway in Glut1‐expressing CD4+ T cells from HIV‐infected individuals who were treatment naive or combination antiretroviral therapy treated (cART‐treated). We also measured the levels of cell‐associated HIV DNA in Glut1+ and Glut1− CD4+ T cells of these individuals. Identification of molecules that regulate key steps in glucose metabolism in CD4+ T cells will improve our understanding of the metabolic pathways that might contribute to HIV disease progression and persistence.

## Methods

### Participant recruitment and blood separation

The main study population included 10 HIV‐infected (HIV+) untreated individuals from the Clinical Research Core Repository at the University of Washington, Seattle, USA, who were subsequently placed on cART. Viable cryopreserved peripheral blood mononuclear cells (PBMCs) (originally collected in EDTA anticoagulant) were shipped in liquid phase nitrogen to Melbourne (Australia) from Seattle. Anthropometric, clinical, and laboratory data were made available for these subjects. Six HIV‐seronegative control subjects and seven HIV+ treatment‐naive subjects were recruited from the Burnet Institute and The Infectious Diseases Unit at The Alfred Hospital, Melbourne, Victoria, Australia. These HIV+ treatment‐naive subjects contributed samples for use in *in vitro* assays only. Informed consent was obtained from all participants, and the research was approved by the University of Washington Ethics Committee and The Alfred Hospital Research Ethics Committee. Fresh blood samples from subjects recruited in Melbourne were collected in citrate or EDTA anticoagulant tubes. Exclusion criteria for participation included co‐infection with hepatitis C virus, active malignancy, vaccination, physical trauma, or surgery within 3 weeks prior to participation. PBMCs from two HIV+ subjects included to enumerate total cellular HIV DNA were obtained from the Immunovirology Research Network repository in Sydney, Australia.

### Flow cytometric analysis

White blood cells in fresh samples were immune‐phenotyped within an hour of collection or cryopreserved as previously described 9, 31. Freshly isolated cells or thawed PBMCs (> 90% viability) were stained on ice for 30 min in the dark using the following pretitrated antibodies: CD3‐APC, CD4‐PerCP, CD8‐PE, CD38‐PE, CCR5‐APC, and HLA‐DR‐FITC (all from BD Biosciences, North Ryde, Australia). Analysis was performed on a FACSCalibur flow cytometer (BD Biosciences). At least 100 000 events were acquired within the lymphocyte gate. flowjo software, version 8.8 (Tree Star, Inc, Ashland, OR, USA) was utilized for data analysis.

### Glucose transporter 1 detection

Cell surface Glut1 expression on freshly isolated or cryopreserved PBMCs was measured by flow cytometry using the Glut1 antibody [MAB1418 clone (R&D Systems, Minneapolis, MN, USA)], as previously described 9. A pilot analysis observing Glut1 expression on T cells revealed that the cryopreservation and thawing process had no effect on Glut1 expression or on the metabolic status of these cells.

### Proliferation assay

PBMCs were resuspended at a concentration of 1 × 10^6^ cells·mL^−1^ in 1 × PBS and incubated at 37 °C for 7 min with 2.5 μm carboxyfluorescein diacetate succinimidyl ester (CFSE; Thermo Fisher Scientific, Waltham, MA, USA). CFSE labeling was terminated by washing the cells three times with cold 1 × PBS/0.5% FCS (v/v). Cells were resuspended in 1 × PBS and analyzed on a FACSCalibur flow cytometer (BD Biosciences).

### Western blot analysis

Samples were lysed and protein concentrations were determined via a bicinchoninic acid protein assay (Thermo Fisher Scientific). Lysates were solubilized and 10 μg protein loaded onto SDS PAGE gel, and Immunoblotting was performed as previously described 32, using primary antibodies specific for phosphorylated Akt (Ser473), and total Akt (all from Cell Signaling Technology, Danvers, MA, USA). Images were detected with enhanced chemiluminescence technique.

### Extracellular flux analysis of glycolytic metabolism

The Seahorse XFe‐24 Extracellular Flux Analyser (Seahorse Biosciences, Billerica, MA, USA) was used to determine the basal rate of glycolysis of cells. Briefly, CD4+ T cells were adhered to the bottom of the wells of a 24‐well Seahorse plate in assay buffer (unbuffered DMEM supplemented with 25 mm glucose and 1 mm sodium pyruvate, pH 7.4) and equilibrated in buffer in a non‐CO_2_ incubator for 60 min prior to assay. The assay protocol consists of repeated cycles of mixing (3 min), incubation (2 min), and measurement (3 min) periods. Readings were taken after 16 min. Extracellular acidification rate (ECAR) was measured by excitation of fluorophores for H^+^, indicative of nonoxidative metabolism.

### HIV infection and DNA amplification

#### Viruses

The CXCR4‐tropic NL4‐3 HIV proviral DNA was obtained through the NIH AIDS Research & Reference Reagent Program (where it was originally deposited by Dr Malcolm Martin) 33. The CCR5‐tropic NL4‐3‐AD8 HIV clone was obtained through the AIDS Research and Reference Reagent Program (originally from Dr Eric O. Freed) 34.

Enhanced green fluorescent protein (EGFP) was inserted into the *nef* open‐reading frame of NL4‐3 or NL4‐3‐AD8 to generate NL4‐3‐Δnef‐EGFP or NL4‐3‐AD8‐Δnef‐EGFP, respectively. The pBR‐NL4‐3‐IRES‐EGFP‐nef+ construct 35 was kindly provided by Dr F. Kirchhoff (University of Ulm, Germany).

#### HIV infection

CD4+ T cells from HIV+/cART subjects were infected with NL4‐3‐Δnef‐EGFP or NL4‐3‐AD8‐Δnef‐EGFP. Virus infectivity was normalized by measuring HIV reverse transcriptase (RT) activity via a micro‐RT assay, as previously described 36. Samples were treated with virus for 2 h at 37°C, washed twice with cold 1 × PBS, and resuspended in RPMI 1640 supplemented with 10% FCS, 2 mm l‐glutamine (Invitrogen), penicillin/streptomycin (100 U·mL^−1^; Invitrogen, Australia), and 5 ng·mL^−1^ of human interleukin‐2 (IL‐2; R&D Systems). Cells were cultured for 3 days, and viral infection was determined by the detection of GFP+ cells within the FL1 channel of a FACSCalibur.

### HIV quantification in CD4+ T cells from treatment‐naive and cART‐treated HIV+ subjects

CD4+ T cells were purified from PBMCs using the Human EasySep CD4+ T cell enrichment kit (Stem Cell, Technology Inc, Vancouver, BC, Canada), and lysates were analyzed for total HIV DNA and integrated DNA using real‐time RT‐PCR as previously described 37.

### Phospho‐flow and intracellular staining

PBMCs were thawed and resuspended in supplemented RPMI‐1640 medium and allowed to rest for 24 h at a concentration of 1 × 10^6^ cell·mL^−1^ at 37 °C, 5% CO_2_. Cells were fixed and permeabilized using the IntraStain Kit (Agilent, Santa Clara, CA, USA), and incubated with anti‐p‐Akt (T308)‐PE antibody (BD Biosciences) following recommended procedures from the manufacturer. The cells were fixed with 0.5% PFA before analysis on a FACSCalibur (BD Biosciences).

### Statistical analysis

Statistical analysis was performed using graphpad prism statistical software (GraphPad Software, San Diego, CA, USA). The nonparametric Mann–Whitney *t*‐test was used for comparison tests of unpaired data and the paired *t*‐test, or the Wilcoxon matched‐pairs signed‐rank test was used to test significance between paired data. Spearman rank test was used for correlation analyses.

## Results

### Subject clinical characteristics

The demographic characteristics and clinical parameters of patients and controls recruited into this study are summarized in Table 1. A total of 16 subjects, including 10 who were HIV+ and treatment naive (HIV+/naive) and subsequently started on combination antiretroviral therapy (HIV+/cART) and six who were HIV seronegative (HIV−), were recruited to evaluate CD4+ T cell Glut1 cell surface expression and intracellular PI3K–Akt signaling in thawed PBMCs. There were no significant differences in body mass index (BMI) or age between the HIV+ and HIV− groups. At the time of recruitment, the median CD4+ T cell count in the HIV+/naive subjects was 223 cells·μL^−1^, and this median count increased to 338 cells·μL^−1^ after 3.3 years (range: 2.1–4.0, *P* = 0.01) on cART.

**Table 1:** Clinical characteristics of study groups

Variables	*n*	Groups			*P* value		
		HIV− (A)	HIV+/naive (B)	HIV+/cART (C)	A vs B	B vs C	A vs C
Sex (M)	26	6	10	10	–	–	–
BMI (kg·m^−2^)	24	23.5 (22.0–25.8)	24.3 (20.4–33.1)	22.7 (18.9–34.7)	0.66	0.82	0.52
Age (years)	26	39.0 (33.0–49.0)	37.0 (28.5–53.2)	40.6 (30.7–55.3)	0.77	**0.002**	0.83
CD4+ T cell count (cells·μL^−1^)	20	–	223 (122.5–379.8)	338 (177.5–575.5)	–	**0.01**	–
Time on cART (years)	10	–	–	3.3 (2.1–4.0)	–	–	–
% CD3+CD4+ T cells	26	53.8 (42.8–63.2)	29.3 (11.6–32.6)	37.2 (26.3–52.8)	**0.0005**	**0.004**	0.11
Viral load (copies·mL^−1^)	20	–	91 900 (37 925–188 000)	< 50	–	–	–

Continuous variables are expressed in median (interquartile range). The nonparametric Mann–Whitney *t*‐test was used to evaluate significant difference between the HIV− group and the others. The Wilcoxon matched‐pairs signed‐rank test was used to evaluate significant differences between the HIV+/naive and HIV+/cART group. Bold numbers represent significant *P* values.

### Glut1 expression is associated with activation and proliferation of CD4+ T cells

**Figure 1:**
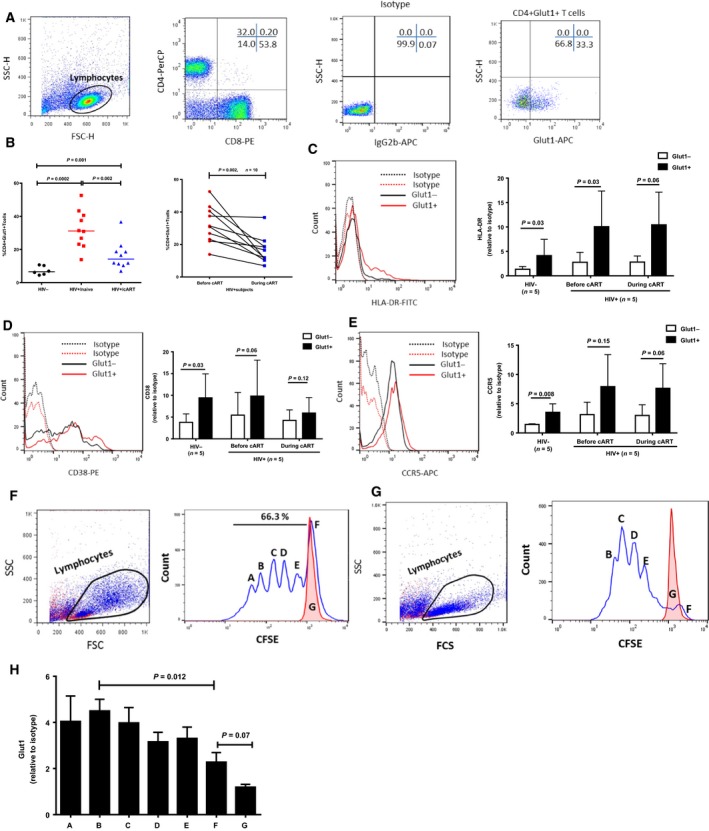
Glut1 cell surface expression on CD4+ T cells is associated with markers of proliferation and activation. (A) Representative flow cytometric dot plots of PBMCs from an HIV+/cART subject. Lymphocytes (circled) were defined using side scatter (SSC) and forward scatter (FSC) characteristics. The gating strategy shows T cells defined based on CD4 and CD8 surface expression. A representative Glut1‐isotype, and Glut1 antibody staining on CD4+ T cells in peripheral blood from HIV+/cART subjects. (B) Percentage of CD4+Glut1+ T cells in peripheral blood from HIV‐negative, HIV+/naive, and HIV+/cART subjects (left panel). Same subjects as in left panel showing percentages of CD4+Glut1+ T cells before and during cART (right panel). (C) Representative histogram (left panel) and aggregate plot (right panel) of HLA‐DR expression on CD4+Glut1+ and CD4+Glut1− T cell from HIV+ subjects. (D) Representative histogram (left panel) and aggregate plot (right panel) of CD38 expression on CD4+Glut1+ and CD4+Glut1− T cell from HIV+ subjects. (E) Representative histogram (left panel) and aggregate plot (right panel) of CCR5 expression on CD4+Glut1+ and CD4+Glut1− T cell from HIV+ subjects. 5 HIV− and 5 HIV+ subjects were analyzed for all surface markers. (F, G) Representative dot plots showing forward and side scatter properties of PBMCs from two HIV‐negative subject stimulated with 10 μg·mL^−1^ PHA plus 5 ng·mL^−1^ IL‐2 for 4 days (blue dots), or cultured without stimulation for the same amount of time (red dots) (left panel). (Right panels) Cells were labeled with CFSE on day 1 as described in Methods, and representative plots of CFSE‐labeled CD4+ T cells after 4 days of incubation with PHA plus IL‐2 (blue line) or unstimulated (red line). (H) The bar chart indicates the cumulative MFI of Glut1 relative to isotype control on CD4+ T cells within each corresponding peak (A–G) showing different rounds of CD4+ T cell replication, and peak G (red) showing CD4+ T cells from unstimulated PBMCs. Cumulative results are obtained from 4 to 5 peaks depending on the amount of cell division. The paired *t*‐test was used to measure significant differences within groups.

We confirmed glucose metabolic activation in our HIV‐positive groups by evaluating Glut1 expression on CD4+ T cells. Figure 1A illustrates the gating strategy used. Similar to previous report 9, the frequency of circulating CD4+Glut1+ T cells was increased in HIV+ subjects and remained elevated above normal levels in cART‐treated individuals (Fig. 1B). To determine if Glut1 expression is associated with activation, we determined the levels of activation markers CD38 and HLA‐DR on CD4+Glut1+ and CD4+Glut1− cells. CD4+Glut1+ T cells from HIV‐negative and HIV‐positive subjects irrespective of treatment status had modestly higher levels of activation markers HLA‐DR (Fig. 1C) and CD38 (Fig. 1D), and the HIV co‐receptor, CCR5 (Fig. 1E) than did CD4+Glut1− T cells. To determine if Glut1 expression is associated with proliferation of CD4+ T cells, Glut1 expression was evaluated over time in dividing CD4+ T cells from uninfected donors. Using CFSE‐labeled PBMCs activated with PHA and IL‐2 for 4 days, higher cell surface Glut1 expression was observed on CD4+ T cells that had undergone proliferation. Figure 1F,G is representative dot plots from four subjects in independent experiments; however, due to variations in the number of peaks in the activated samples, the number of data points per peak in the aggregate graph (Fig. 1H) varied from 5 to 6 (Fig. 1F,G, right panels). Collectively, these data suggest that Glut1 expression is associated with CD4+ T cell activation and proliferation.

### The PI3K pathway is activated in CD4+Glut1+ T cells

**Figure 2:**
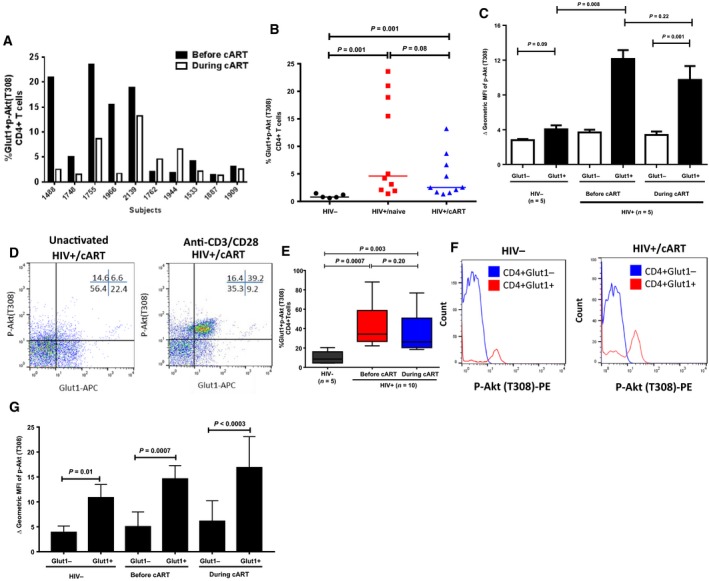
Glut1 cell surface expression on CD4+ T cells is associated with high PI3K activity. (A) Individual comparisons of changes in the percentage of CD4+Glut1+p‐Akt (T308)+ T cells in peripheral blood of HIV+ subjects before and during cART. (B) Aggregate percentage of CD4+Glut1+p‐Akt (T308)+ T cells in peripheral blood from HIV−, and HIV+ subjects before and during cART. (C) Geometric MFI of p‐Akt (T308) in Glut1− and Glut1+CD4+ T cells in PBMCs from HIV−, and HIV+ subjects before and during cART. (D) Representative dot plots showing percentage of CD4+Glut1+p‐Akt (T308)+ T cells within the CD4+ T cell populations of PBMCs from HIV+ subjects stimulated with anti‐CD3/28 microbeads, and (E) showing cumulative data. (F) Representative histogram showing the shift in fluorescence intensity of p‐Akt (T308)‐PE in CD4+ T cell compartments in PBMCs from HIV‐negative and HIV+/cART subjects stimulated with anti‐CD3/28 microbeads, with cumulative data represented in (G). The error bars represent mean (SEM). The paired *t*‐test and the Mann–Whitney *t*‐tests were used to measure significant differences within and between the groups, respectively.

Activation of T cells induces PI3K activity that is involved in Glut1 translocation from the cytoplasm to the cell surface membrane 38. Utilizing phospho‐flow technology, the percentage of CD4+Glut1+ T cells expressing p‐Akt (T308), a downstream effector of the PI3K pathway, was found to be elevated in HIV+/naive subjects compared with HIV− controls (Fig. 2A,B). Notably, CD4+Glut1+ T cells from HIV+/naive and HIV+/cART subjects expressed significantly higher p‐Akt (T308) than did CD4+Glut1+ T cells from HIV− subjects (Fig. 2C). Furthermore, CD4+ T cells from HIV+ subjects expressed significantly higher levels of p‐Akt (T308) in response to anti‐CD3/CD28 microbeads co‐stimulation than did CD4+ T cells from HIV− individuals, illustrating a hypermetabolic response (Fig. 2D,E).

We followed up these observations and showed that, during *in vitro* activation of PBMCs from HIV− and HIV+/cART subjects, CD4+Glut1+ T cells expressed higher levels of p‐Akt (T308) than did CD4+Glut1− T cells (Fig. 2F,G), consistent with higher PI3K activity.

### CD4+Glut1+ T cells from patients are not enriched for total HIV DNA

**Figure 3:**
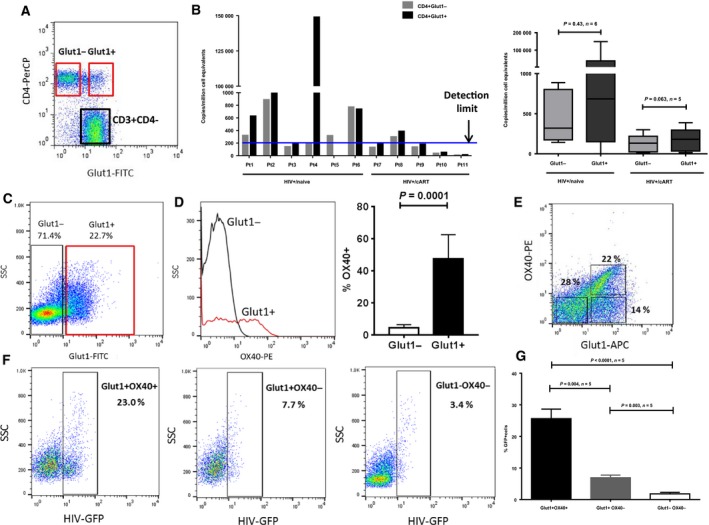
CD4+Glut1+ cells take up more HIV in culture than do CD4+Glut1− T cells. (A) Representative flow cytometric dot plot showing the cell surface expression of Glut1 on CD4+ T cells after being gated within the lymphocyte and CD3+ T cell population of PBMCs from HIV+/naive or HIV+/cART subjects. The red square represents the gating strategy used to sort Glut1− from Glut1+ cells within the CD4+ population in order to analyze total cellular HIV DNA. (B, Left panel) Total HIV DNA in CD4+Glut1+ and CD4+Glut1− T cell populations in HIV+/naive (*n* = 6) and HIV+/cART (*n* = 5) subjects. The blue line represents the detection limit of the assay. (B, Right panel) Combined data for total HIV DNA levels in CD4+Glut1+ and CD4+Glut1− T cells from HIV+/naive or HIV+/cART subjects. (C) Representative dot plot of cells gated on CD3+CD4+ T cells from PBMCs of HIV+/cART subjects cultured for 3 days in the absence of GFP‐tagged HIV (*n* = 5). (D) Representative histogram of OX40 on CD4+Glut1− and CD4+Glut1− cells from subjects in C (left panel), with cumulative data shown (right panel). (E) Representative dot plot showing OX40 and Glut1 expression on cells gated on CD4+ T cells from PBMCs of HIV+/cART subjects cultured for 3 days with GFP‐labeled HIV. (F) Representative dot plots showing the percentages of GFP+ cells in CD4+Glut1+OX40+, CD4+Glut1+OX40−, and CD4+Glut1−OX40− T cells. (G) Graph showing cumulative frequency of GFP+ cells within different populations of CD4+ T cells based on their cell surface expression of Glut1 and OX40 (*n* = 5). The Wilcoxon matched‐pairs signed‐rank *t*‐test was used to evaluate significant differences between the levels of HIV DNA in CD4+Glut1+ and CD4+Glut1− T cells in HIV+/cART subjects. The Mann–Whitney *t*‐tests were used to measure significant differences in the percentage of GFP+ cells within the CD4+ T cell populations.

Since increased Glut1 expression on CD4+ T cells has been shown to facilitate HIV infection 11, CD4+ T cells from HIV+/naive and HIV+/cART subjects were sort‐purified into CD3+CD4+Glut1− and Glut1+ subpopulations (Fig. 3A) and examined for total HIV DNA content. CD3+CD4− T cells represent CD8+ T cells which we have previously shown to express high basal Glut1 levels 9. This contrasts results from other groups who have reported that Glut1 is undetectable or is expressed at low levels in quiescent T cells 12, 39, 40. Thus, our results herein call into question the interpretation of data obtained using different Glut1 detection techniques, or the specificity of the Glut1 antibody MAB1418 clone (R & D Systems), at least for CD8+ T cells.

Although the total HIV DNA content was higher in CD4+Glut1+ T cells than in Glut1− cells from some patients, this was not universal (Fig. 3B), suggesting that Glut1 expression on CD4+ T cells might be driven by other factors independent of direct HIV infection, such as inflammatory cytokines as previously suggested 38. Of note, there were no clinically significant differences between Pt4 and the other subject in this analysis. The percentage of CD4+Glut1+ T cells for each subject is shown in Table S1.

### Total HIV is predominantly found within CD4+Glut1+OX40+ T cells *in vitro*

To determine whether levels of activation in unstimulated CD4+Glut1+ T cells support HIV infection and replication, we selected PBMCs from five virologically suppressed HIV+/cART subjects who had a high percentage of CD4+Glut1+ T cells, four of whom had undetectable cellular HIV DNA, and exposed them to a GFP‐tagged HIV in the absence of exogenous activating stimuli. Since PI3K activity and Glut1 are reportedly important for HIV infection *in vitro* 11, we reasoned that CD4+Glut1+ T cells from those patients with high PI3K activity might be preferential targets of HIV *in vitro*. Figure 3C illustrates a dot plot of CD4+Glut1− and CD4+Glut1+ T cells in PBMCs from an HIV+/cART subject cultured for 3 days in the absence of GFP‐tagged HIV. Noticeably, OX40 expression was greater on Glut1‐positive cells than on Glut1− cells (Fig. 3D). Examining OX40 expression on CD4+ T cells in HIV‐negative and HIV+/cART subjects, we observed increased frequency of CD4+OX40+ T cells in anti‐CD3/CD28‐activated cells (*P* = 0.03; Fig. S1).

OX40 (CD134) is a member of the tumor necrosis factor receptor superfamily, and its expression is a surrogate marker of PI3K activity in T cells 41; thus, we anticipated that Glut1+OX40+ cells would be preferentially infected by HIV *in vitro*. Figure 3E illustrates that the majority of OX40+ cells express Glut1. HIV‐GFP+ cells were enriched within the CD4+Glut1+OX40+ T cell population, with significantly less infection in CD4+Glut1−OX40− cells (Fig. 3F,G). For this specific experiment, we were unable to include a live/dead marker in this panel due to accessibility of only a four color FACS instrument. However, we are confident that most of the gated cells were viable because, prior to staining, viability was > 95% as assessed by trypan blue staining. Together, these *ex vitro* data suggest that basal expression of Glut1 and P13K signaling in virologically suppressed cART‐treated HIV+ subjects represented a sufficiently activated cellular state permissible to HIV infection.

### Inhibition of PI3K subunit, p110γ, suppresses HIV infection of CD4+ T cells in cell culture

**Figure 4:**
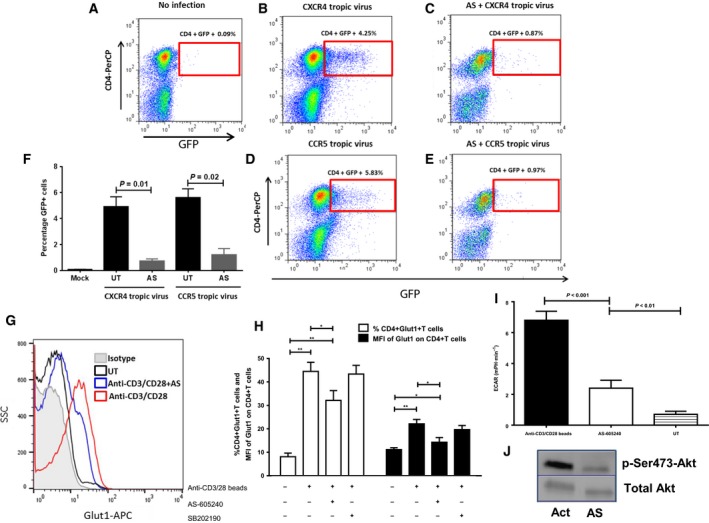
Inhibition of PI3Kγ in CD4+ T cells suppresses glycolysis and HIV infection. Purified PBMCs for HIV− subjects were pretreated for 48 h with 200 nm AS‐605240 (AS) and activated with 10 μg·mL^−1^ PHA plus 5 ng·mL^−1^ IL‐2 for 24 h prior to infection with either a CXCR4 or a CCR5 tropic virus. (A) PBMCs were activated but were not incubated with HIV. (B) PBMCs with no inhibitor prior to activation, incubated with CXCR4 tropic HIV. (C) PBMCs pretreated with AS‐605240 (AS) prior to activation and incubated with CXCR4 tropic HIV. (D) PBMCs with no inhibitor prior to activation, incubated with CCR5 tropic virus. (E) PBMCs pretreated with AS‐605240 (AS) prior to activation and incubated with CCR5 tropic virus. (F) Combined data from three independent experiments showing the effects of PI3K subunit p110γ isoform inhibition on HIV infection. The Wilcoxon matched‐pairs signed‐rank *t*‐test was used to evaluate significant differences between treatments. (G, H) Negatively selected and purified CD4+ T cells from HIV− controls were left untreated or treated with AS for 24 h followed by activation with anti‐CD3/CD28 beads (bead: cell ratio, 1 : 2), and Glut1 cell surface expression measured. Representative histogram is indicated in panel G and shows the effect of AS on Glut1 expression. Activated cells were stained with isotype control. Cumulative data are shown in panel H. The p38 MAP kinase inhibitor SB202190 (10 μm) was used as a nonmetabolic inhibitor control**.** (I) ECAR of purified CD4+ T cells. Cells were either left unactivated (UT), or inhibited and activated as in G above. Bars graphs represent mean ± SD. Differences between individual groups were analyzed using the nonparametric two‐tailed Mann–Whitney *U* test. Statistical differences are indicated by *P* values above the plots. (J) Western blot analysis showing the effect of AS‐605240 pretreatment on PI3K activity (p‐Ser473Akt) in anti‐CD3/CD28‐activated (Act) CD4+ T cells. Total Akt was used as a reference control.

We next wanted to determine whether pretreatment of PBMCs from uninfected subjects with AS‐605240 (AS), a specific inhibitor of the PI3K p110γ isoform 42, 43, 44, would affect HIV infection. Treatment of PBMCs with AS reduced infection of CD4+ T cells by either CXCR4‐tropic (Fig. 4A–C,F) or CCR5‐tropic viruses (Fig. 4A,D–F). To confirm the metabolic effects of AS, CD4+ T cells were activated in the presence or absence and absence of AS and Glut1 expression and glycolysis evaluated. We found that AS modestly suppressed both Glut1 expression on CD4+ T cells (Fig. 4G,H) and the ECAR (indicative of glycolysis; Fig. 4I). Furthermore, phosphorylation of Ser473‐Akt (Fig. 4J), which is essential for activation of the PI3K–Akt pathway, was suppressed but not completely abrogated by AS treatment 45. Collectively these data suggest that the PI3Kγ partially controls the glucose metabolic components of CD4+ T cells essential for efficient HIV infection 11, 46.

## Discussion

We have previously shown that the proportion of CD4+ T cells that express Glut1 is significantly increased in HIV‐infected individuals and is associated with increased glucose uptake and lactate production by CD4+ T cells, as well as CD4+ T cell depletion *in vivo* 9. Here, we investigated a candidate Glut1 regulating pathway, PI3K–Akt, to evaluate its potential role in the regulation of Glut1 on CD4+ T cells in HIV‐infected subjects. We report a hyperresponsive PI3K signaling in CD4+ T cells from HIV+ subjects associated with increased phosphorylation of Akt (T308). *In vitro* experiments also showed that a Glut1+OX40 phenotype renders CD4+ T cells from cART‐treated HIV+ subjects permissive to HIV infection without external activating stimuli. CD4 infectivity by HIV was sensitive to PI3Kγ inhibition.

Our work corroborates previous findings by Hegedus *et al*. and Loisel‐Meyer *et al*. 11, 47 based on *in vitro* observations that Glut1‐mediated glucose metabolic pathways are critical regulators of HIV infection in T cell lines and primary CD4+ T cells 1, 11, reflective of an altered metabolic profile favoring viral infection and replication 1. Recently, it has been hypothesized that hyperactivation of aerobic glycolysis pathways in CD4+ T cells during HIV infection could foster apoptosis and accelerate their destruction 46, 48. We extended this observation to demonstrate the involvement of the specific PI3Kγ in metabolic activation and permissiveness of CD4+ T cells to HIV. Furthermore, in the absence of external stimuli, we identified a population of CD4+Glut1+OX40+ cells that are preferentially infected. Noteworthy, the overwhelming majority of CD4+OX40 cells co‐express Glut1, suggesting that OX40 expression reflects a metabolic signature permissive to HIV infection.

The finding of heightened expression of the high affinity glucose transporter Glut1 on CD4+ T cells corroborates previous studies, which demonstrated that HIV infection *in vitro* was associated with elevated glucose uptake and increased glycolysis 1, 2, 47. Similar findings have been observed in infection with polyoma virus 49 and HCMV 4, 50. Our data demonstrate that CD4+Glut1+ T cells from patients were highly susceptible to HIV infection *in vitro* without requiring additional activating stimuli. Unexpectedly, although there were marginally increased HIV DNA in sorted CD4+Glut1+ T cells compared with CD4+Glut1− T cells in some HIV+ subjects, this was not universal and cumulative data showed no significant differences. It is possible that HIV is confined to distinct subpopulations of CD4+Glut1+ T cells, such as resting memory T cells that express Glut1. Subjects with a high percentage of CD4+Glut1+ T cells normally have a low CD4 T cell count 9 and, given constraints on the volume of blood that could be drawn under the ethical guidelines, we were unable to acquire sufficient numbers of cells to analyze specific subpopulations of Glut1‐expressing CD4+ T cells. Efforts to measure HIV protein in CD4+ T cells from HIV+ subjects in the absence of stimulation were also not forthcoming. Thus, a limitation of this study was the inability to precisely characterize HIV levels in specific subpopulations of CD4+Glut1+ and CD4+Glut1− T cells. Such characterization will broaden our understanding of HIV pathogenesis including the potential role of glycolytic metabolism and PI3K‐mTOR signaling in HIV latency in specific CD4+ T cell subpopulations. Of note, we found high levels of Glut1 binding on CD8+ T cells using the MAB1418 antibody clone from R&D systems. This contradicts other groups who have reported the absence of Glut1 on quiescent CD8+ T cells 12, 39, 40. In a previous study, we found that CD4+ T cells reactive to the MAB1418 clone took up more glucose and produced more lactate compared with nonreactive cells 9. This suggests that the MAB1418 clone may at least in part define a state of high metabolic activity in activated CD4+ T cells. However, since several authors reported that Glut1 is not expressed on resting CD8+ T cells, this controversy warrants clarification and remains to be resolved in future studies.

Phospho‐flow analysis showed a high level of phosphorylation of Akt at T308 (indicative of PI3K activation) in CD4+Glut1+ T cells, demonstrating that PI3K–Akt pathway contributes at least partly to the increased glucose metabolism in CD4+ T cells in HIV+ individuals and corroborating previous findings that activation of the PI3K–Akt pathway is considered an important switch to activate metabolic programs characteristic of activated and proliferating T cells 22, 51, 52. An interesting novel observation is that CD4+ T cells from HIV+ subjects exhibit PI3K‐directed metabolic hyperactivation, a phenomenon similar to that described by monocytes rechallenged with *Candida albicans* cell wall constituent β‐glucan, termed monocyte memory or trained immunity 53.

In *in vitro* experiment described by Loisel‐Meyer *et al*. 11, inhibition of cytokine‐induced Glut1 expression and PI3K activation on CD4+T cells by the nonisoform and non‐tissue‐specific PI3K inhibitor, LY294002 54, 55, caused complete abrogation of single‐round HIV infection. We have extended these observations and showed that inhibition of the specific PI3K isoform, PI3Kγ, prior to HIV exposure significantly reduced HIV infection of CD4+ T cells, an observation that may be explained by the requirement of PI3Kγ kinase activity for efficient Akt phosphorylation/activation, optimal T cell activation, and cell cycle progression 26. Furthermore, we observed that PI3Kγ inhibition using AS‐605240 modestly suppressed Glut1 on CD4+ T cells. This is in line with the western blot analysis which showed that PI3Kγ inhibition did not completely abrogate PI3K–Akt signaling as measured by p‐Akt(Ser473). This contrasts results from Barata *et al*. 45 who demonstrated complete abrogation of Akt(Ser473) phosphorylation and total Glut1 in IL‐7 treated TAIL7 cell lines exposed to LY294002. With inference from above, LY294002 may nonspecifically inhibit other PI3K isoforms or networks that also regulate Glut1 cell surface expression on CD4+ T cells. Furthermore, the choice of cell types, activating stimuli, activation status, and concentration of inhibitors distinguished our work from those described above. In closer agreement with our findings, it has been shown that LY294002, PP242 (PI3K/mTOR kinase inhibitor), and rapamycin (mTORC1 inhibitor) suppressed, but did not abrogate the anti‐CD3/CD28‐induced cell surface Glut1 expression in T cells 10. Taken together our data suggest that therapeutic targeting of the PI3Kγ isoform in CD4+ T cells is likely to be more specific since this isoform is preferentially expressed in leukocytes 26, 27, 28.

It may seem counterintuitive that T cells that have a markedly increased demand for energy would be involved in exploiting a relatively inefficient means like glycolysis to generate ATP. However, the shift from oxidative phosphorylation to aerobic glycolysis by rapidly proliferating T cells diverts the use of glucose carbon for macromolecular biosynthesis 46, 52, 56. Thus, it has been proposed that increased glycolysis in CD4+ T cells may render them preferential targets for HIV infection 46. Interestingly, PI3K signaling is tightly coupled to mTOR activity, recently shown to regulate HIV latency 57 and control production of pro‐inflammatory cytokines from activated CD4+ T cells 58. Indeed, inhibition of PI3K and mTOR1/2 has been shown to suppress HIV reactivation and replication, including multidrug resistant strains by reducing cellular biosynthesis 59, 60. Our findings support therapeutic targeting metabolism through the OX40‐PI3Kγ axis in CD4+ T cells to reduce metabolic activity necessary for HIV reactivation and homeostatic proliferation of the CD4+ T cell‐restricted HIV reservoir. Furthermore, the observation that CD4+ T cells from cART‐treated HIV positive individuals are metabolically hyperactive supports the ‘block and lock’ 57 and ‘starve’ 15 HIV cure strategies recently proposed.

In summary, we highlighted for the first time a hyperactive glucose metabolism in CD4+ T cells, and the existence of a population of metabolically active CD4+OX40+ T cells from cART‐treated HIV+ patients highly susceptible to HIV infection. PI3Kγ‐directed inhibition may promote targeted reduction of CD4+ T cell metabolic activation in HIV infection and other inflammatory conditions associated with CD4+ T cells hypermetabolic responses.

## Authors’ contribution

CSP conceived, participated in the conceptual design of the project, conducted the experiments, analyzed data and helped drafted the manuscript. SMC and JMM critically revised the manuscript, participated in data interpretation and conceptual discussions. GAD conducted experiments and analyzed data. MO designed and conducted experiments, interprets data, participated in conceptual discussions, and wrote sections of the manuscript. MCEW helped draft manuscript and participated in conceptual discussions. DCH and SS conducted experiments and analyzed and interpret data. CP and JZ conducted experiments and assisted in the experimental designs. DS and SRL reviewed manuscript and provided intellectual support. All authors read and approved the final manuscript.

## Supporting information


**Fig. S1.** TCR activation induces OX40 expression.

**Table S1.** Percentage of circulating CD4+Glut1+ T cells in participants.
