# Supplementary figures and images for: Metabolically active CD4+ T cells expressing Glut1 and OX40 preferentially harbor HIV during in vitro infection
Source: FEBS Lett. 2017 Oct 11;591(20):3319–32. doi: 10.1002/1873-3468.12843 (PMC5658250; doi:10.1002/1873-3468.12843)

## Slide 1
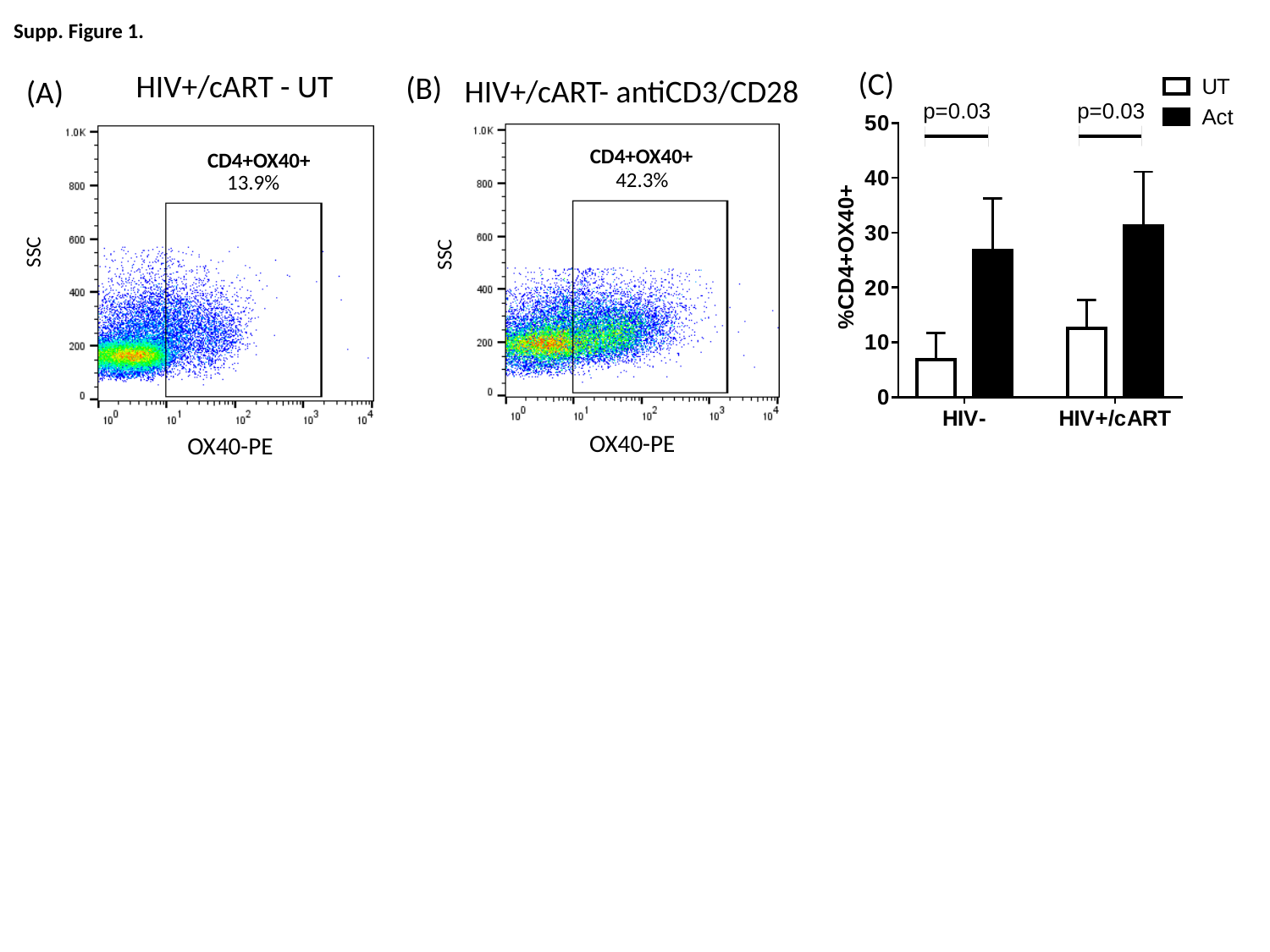

Supp. Figure 1.
(C)
HIV+/cART - UT
(B)
HIV+/cART- antiCD3/CD28
(A)
CD4+OX40+
CD4+OX40+
42.3%
13.9%
 SSC
 SSC
OX40-PE
OX40-PE

Supplement: Supplementary file 1 — Fig. S1. TCR activation induces OX40 expression. [file FEB2-591-3319-s001.pptx]
