# Supplementary material for: Metabolically active CD4+ T cells expressing Glut1 and OX40 preferentially harbor HIV during in vitro infection
Source: FEBS Lett. 2017 Oct 11;591(20):3319–32. doi: 10.1002/1873-3468.12843 (PMC5658250; doi:10.1002/1873-3468.12843)
Supplement: Supplementary file 2 — Table S1. Percentage of circulating CD4+Glut1+ T cells in participants. [file FEB2-591-3319-s002.docx]

Supplementary Table 1: Percentage of circulating CD4+Glut1+ T cells in participants

| **Samples** | **Patient codes** | **% CD4+Glut1+ cells** |
| --- | --- | --- |
| Pt1 | 0001 | 18.3 |
| Pt2 | 0005 | 20.8 |
| Pt3 | LP58 | - |
| Pt4 | LP3 | - |
| Pt5 | 00017 | 16.0 |
| Pt6 | 00029 | 9.5 |
| Pt7 | 00030 | 16.5 |
| Pt8 | 00031 | 13.3 |
| Pt9 | 00032 | 16.8 |
| Pt10 | 00033 | 10.5 |
| Pt11 | 00034 | 12.8 |
